# 2185. *In vitro* activity of aztreonam-avibactam (ATM-AVI), cefiderocol (FDC), and cefepime-taniborbactam (FEP-TAN) against multi-species, NDM-producing *Enterobacterales* causing a local outbreak

**DOI:** 10.1093/ofid/ofad500.1807

**Published:** 2023-11-27

**Authors:** Ellen G Kline, Kevin M Squires, Ghady Haidar, Graham Snyder, Lee Harrison, Daria Van Tyne, Ryan K Shields

**Affiliations:** University of Pittsburgh, Pittsburgh, Pennsylvania; University of Pittsburgh, Pittsburgh, Pennsylvania; University of Pittsburgh School of Medicine, Pittsburg, PA; University of Pittsburgh Medical Center, Pittsburgh, Pennsylvania; University of Pittsburgh, Pittsburgh, Pennsylvania; University of Pittsburgh School of Medicine, Pittsburg, PA; University of Pittsburgh, Pittsburgh, Pennsylvania

## Abstract

**Background:**

New Delhi metallo-β-lactamase (NDM)-producing *Enterobacterales* are increasing in the US. Recommended treatment (tx) options include ceftazidime-avibactam (CZA) plus ATM or FDC. We assessed the *in vitro* activity of tx options in the setting of a multi-species NDM outbreak.

**Methods:**

Patient isolates were identified through active carbapenemase testing and characterized by whole-genome sequencing. Minimum inhibitory concentrations (MICs) for ATM, ATM-AVI, ceftazidime, CZA, FDC, FDC-AVI, FEP and FEP-TAN were determined by broth microdilution. AVI and TAN were tested at 4 mg/L. Index isolates were defined as the first NDM-producing isolate per species per patient.

**Results:**

48 isolates from 24 patients were included from 2018-2023; 73% of isolates were collected in the last year. 17% of patients were infected with >1 NDM-producing species. Among index isolates (n=29; **Table 1**) comprising 10 species, the most common species were *E. cloacae* complex (15), *E. coli* (3), and *K. aerogenes* (3). Isolates harbored NDM-5 (72%) or NDM-1 (28%). Other β-lactamases included ACT (15), SHV (7), TEM (7), and CTX-M (6) variants. 93%, 93%, and 69% of index isolates were susceptible to ATM-AVI, FEP-TAN, and FDC, respectively; corresponding MIC_50_ values were 0.06, 0.5, and 2 mg/L, respectively (**Table 2**). FDC susceptibility improved to 86% with addition of AVI.

Among serial isolates (n=19), rates of susceptibility were 89%, 84%, and 16% for ATM-AVI, FEP-TAN, and FDC, respectively (**Table 3**). Two patients were treated with CZA-ATM. In 1 case ATM-AVI MICs increased against *E. coli* from 16 to >128 mg/L post-CZA-AVI tx, attributed to an insertion (YRIN) and substitution (A417V) in *ftsI* (PBP3). Overall, 4 isolates (all *E. coli*) from 2 patients were non-susceptible to ATM-AVI, FEP-TAN, and FDC. All were related by WGS and harbored CMY-145, NDM-5, and a YRIN duplication in PBP3.

**Figure:**
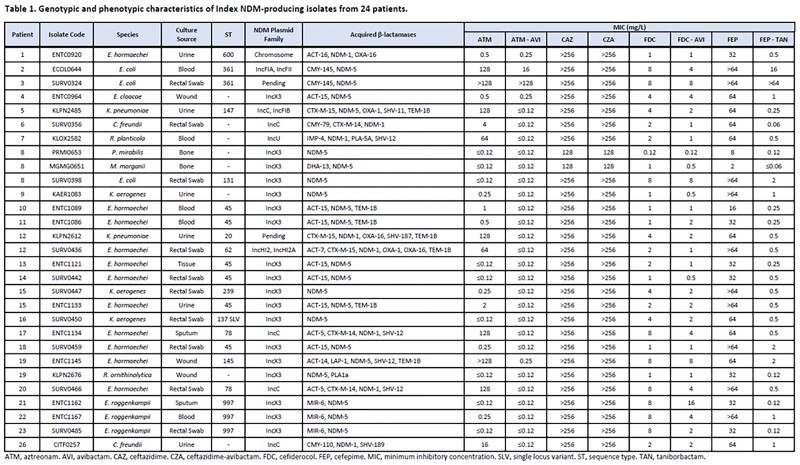

**Figure:**
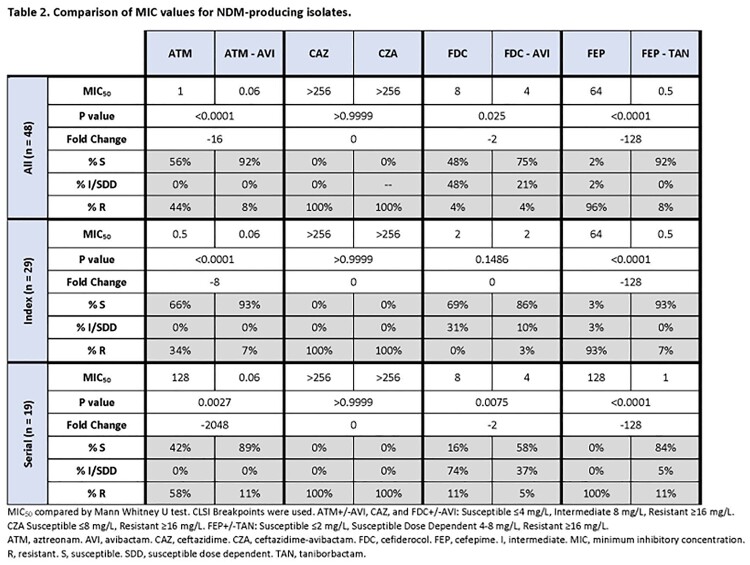

**Figure:**
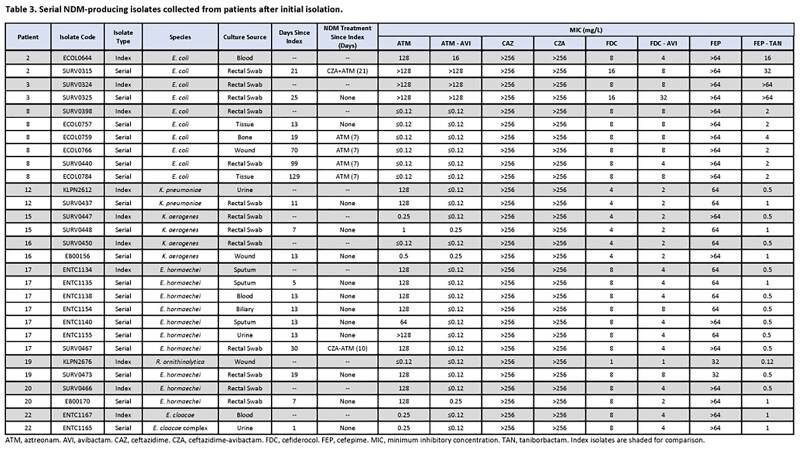

**Conclusion:**

During a multi-species NDM outbreak we found ATM-AVI and FEP-TAN demonstrated potent *in vitro* activity against clinical isolates. Susceptibility rates for both β-lactam/β-lactamase inhibitors was higher than FDC, suggesting they may be preferred for tx in the absence of timely susceptibility testing. The activity of each agent is compromised by NDM-producing strains that harbor PBP3 mutations.

**Disclosures:**

**Ghady Haidar, MD**, Allovir: Grant/Research Support|AstraZeneca: Advisor/Consultant|AstraZeneca: Grant/Research Support|Karius: Advisor/Consultant|Karius: Grant/Research Support|NIH: Grant/Research Support **Ryan K. Shields, PharmD, MS**, Allergan: Advisor/Consultant|Cidara: Advisor/Consultant|Entasis: Advisor/Consultant|GSK: Advisor/Consultant|Melinta: Advisor/Consultant|Melinta: Grant/Research Support|Menarini: Advisor/Consultant|Merck: Advisor/Consultant|Merck: Grant/Research Support|Pfizer: Advisor/Consultant|Roche: Grant/Research Support|Shionogi: Advisor/Consultant|Shionogi: Grant/Research Support|Utility: Advisor/Consultant|Venatorx: Advisor/Consultant|Venatorx: Grant/Research Support

